# Increased cortical structural covariance correlates with anhedonia in schizophrenia

**DOI:** 10.1038/s41537-023-00350-3

**Published:** 2023-04-04

**Authors:** Lingfang Yu, Zenan Wu, Dandan Wang, Chaoyue Guo, Xinyue Teng, Guofu Zhang, Xinyu Fang, Chen Zhang

**Affiliations:** 1 Schizophrenia Program, Shanghai Mental Health Center, Shanghai Jiao Tong University School of Medicine, Shanghai, 200030 China; 2 The Affiliated Wuxi Mental Health Center of Jiangnan University, Wuxi, 214151 China; 3 The Affiliated Brain Hospital of Nanjing Medical University, Nanjing, 210029 China

**Keywords:** Schizophrenia, Prefrontal cortex

## Abstract

Anhedonia is a common symptom in schizophrenia and is closely related to poor functional outcomes. Several lines of evidence reveal that the orbitofrontal cortex plays an important role in anhedonia. In the present study, we aimed to investigate abnormalities in structural covariance within the orbitofrontal subregions, and to further study their role in anticipatory and consummatory anhedonia in schizophrenia. T1 images of 35 schizophrenia patients and 45 healthy controls were obtained. The cortical thickness of 68 cerebral regions parcellated by the Desikan-Killiany (DK) atlas was calculated. The structural covariance within the orbitofrontal subregions was calculated in both schizophrenia and healthy control groups. Stepwise linear regression was performed to examine the relationship between structural covariance and anhedonia in schizophrenia patients. Patients with schizophrenia exhibited higher structural covariance between the left and right medial orbitofrontal thickness, the left lateral orbitofrontal thickness and left pars orbitalis thickness compared to healthy controls (*p* < 0.05, FDR corrected). This results imply that the increased structural covariance in orbitofrontal thickness may be involved in the process of developing anhedonia in schizophrenia. The result indicated that the increased structural covariance between the left and right medial orbitofrontal thickness might be a protective factor for anticipatory pleasure (*B’* = 0.420, *p* = 0.012).

## Introduction

Schizophrenia is a severe mental disorder that affects 0.5–1% of the population worldwide^1^, and mainly manifests as positive symptoms, negative symptoms, and cognitive impairments. Negative symptoms tend to be residual after treatment and are associated with poor functional outcomes of schizophrenia^2,3^. Anhedonia is one of the most common negative symptoms in schizophrenia, with an estimated prevalence rate arranging from 45 to ~80%^4,5^. Anhedonia refers to the reduced capacity to experience pleasure in activities that individuals would normally enjoy^6^ and is considered to be a vital aspect of psychiatric disorders. The Measurement and Treatment Research to Improve Cognition in Schizophrenia (MATRICS) consensus conference on negative symptoms suggests that anhedonia is one of the five categories of negative symptoms in schizophrenia^7^. In the “Diagnostic and Statistical Manual of Mental Disorders, Fifth Edition” (DSM-5), anhedonia is defined as “Lack of enjoyment from, engagement in, or energy for life’s experiences; deficits in the capacity to feel pleasure and take interests in things.” Anhedonia has also been divided into consummatory anhedonia and anticipatory anhedonia based on the components of pleasure^8^. The former refers to the reduction in experiencing pleasure when engaging in an enjoyable activity, while the latter refers to the reduction in experiencing pleasure related to future activities. It was assumed that the two different components of anhedonia may involve distinct biological mechanisms. The presence of anhedonia usually suggests an unfavorable situation, such as treatment resistance and low quality of life in patients with depression^9^, and poor outcomes and disability in patients with schizophrenia^10^. Moreover, anhedonia may be responsible for a higher risk of suicidal ideation and suicide attempts in schizophrenia^11,12^. It was also observed that social anhedonia is closely related to poorer neurocognitive functioning^13^. This highlights a crucial need to elucidate how anhedonia develops.

The literature, while still incomplete, has revealed, part of the biological underpinnings of anhedonia. Research into the mechanisms underlying anhedonia plays an important role in potentially mapping the brain abnormalities in anhedonia. Recently, evidence has emerged that the orbitofrontal cortex (OFC) is linked to anhedonia^14^. The OFC is located on the ventral side of the frontal lobe and receives projections from visual, olfactory, taste, and somatosensory regions^15^. Evidence has shown that the OFC and the anterior cingulate gyrus are both related to the pleasure sensation brought by taste^16^. It has been reported that higher levels of physical anhedonia resulted in atypical OFC sulcogyral patterns^17^, whose activity was found to be negatively correlated with anhedonia^14^. Evidence indicates that different subregions of the OFC are involved. The medial orbitofrontal cortex (mOFC) is responsible for measuring the reward value of stimuli, while the lateral orbitofrontal cortex (lOFC) is responsible for measuring the punishment component in ongoing activities and provides a basis for a change in behavior^18^. However, the role of the OFC in reward has mainly been studied in patients with depression. Evidence in schizophrenia patients is relatively rare. Given that many other brain regions have also been reported to be involved in anhedonia, the relationship of the intercortical region may provide new insights into the mechanism of anhedonia.

Structural covariance analysis is an important approach for mapping intercorrelation between brain regions and is promising for investigating neurodevelopmental abnormalities. There is evidence that significant differences in structural covariance exist between schizophrenia patients and healthy controls^19^. However, whether structural covariance alteration contributes to the pathology of anhedonia is still unclear. Given that human functional magnetic resonance imaging (MRI) studies and animal studies have emphasized the crucial role of cortical regions, especially frontal regions in anhedonia, we hypothesized that anhedonia is related to structural covariance within the OFC. The present study set out to delineate the cortical region abnormalities within the OFC related to anhedonia in the construct of consummatory and anticipatory pleasure in schizophrenia using structure covariance analysis.

## Results

### Demographic and clinical variables

A total of 80 subjects were included (35 patients with schizophrenia and 45 healthy controls). There were no significant differences in age, sex, marital status, and education level (*p* > 0.05) between the schizophrenia group and the healthy control group. The schizophrenia group showed significantly lower score of the Temporal Experience of Pleasure Scale (TEPS) (*p* < 0.05). The results are present in Table 1.

**Table 1:** Demographic and clinical information.

	Schizophrenia group (*n* = 35)	Control group (*n* = 45)	*t*/*χ*^2^	*p*
Age/year	30.54 ± 9.590	28.18 ± 6.627	1.303	0.196
Sex/%(*n*)			0.470	0.497
Male	34.29(12)	42.22(19)		
Female	61.71(23)	57.78(26)		
Marital status/%(*n*)			0.954	1
Unmarried	62.86(22)	62.22(28)		
Married	37.14(13)	37.78(17)		
Divorced	0(0)	0(0)		
Widowed	0(0)	0(0)		
Education level/year	13.14 ± 3.751	14.20 ± 2.897	−1.423	0.159
Duration of illness/month	44.37 ± 37.505			
Duration of antipsychotic medication/month	16.11 ± 9.640			
Total antipsychotic dose (olanzapine equivalent)/mg	5721.43 ± 3451.370			
PANSS				
Positive subscale	16.09 ± 6.122			
Negative subscale	16.06 ± 5.228			
General psychopathology subscale	33.68 ± 8.745			
PANSS total score	65.82 ± 16.484			
TEPS				
TEPS-ANT	37.37 ± 8.044	47.07 ± 6.576	−5.931	0.000
TEPS-CON	32.49 ± 7.298	39.56 ± 8.864	−3.817	0.000
TEPS total score	69.86 ± 13.597	85.73 ± 11.604	−5.630	0.000

*PANSS* positive and negative syndrome scale, *TEPS* Temporal Experience of Pleasure Scale, *TEPS-ANT* anticipatory pleasure subscale of TEPS, *TEPS-CON* consummatory pleasure subscale of TEPS.

### Structural covariance of brain regions within the OFC in patients with schizophrenia and healthy controls

**Fig. 1:**
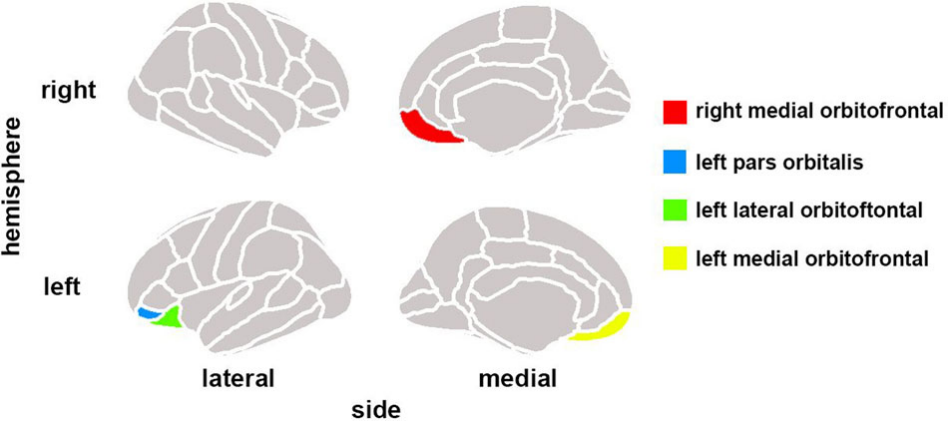
Results. Cortical regions that manifest increased structural covariance in schizophrenia patients compared to healthy controls.

In schizophrenia group, 13 brain structural covariance were significant. In healthy control group, there were 3 brain structural covariance being significant. Finally, 13 brain structural covariance were selected for between-group analysis (see more details in Supplementary Material A). Significantly increased structural covariance between the left and right mOFC thickness (*p* = 0.012, FDR-corrected) and the left lOFC thickness and left pars orbitalis cortical thickness (*p* = 0.032, FDR-corrected) were found in patients with schizophrenia (see more details in Fig. 1 and Table 2).

**Table 2:** Between group differences in structural covariance within the OFC.

Structural covariance	*z* value	FDR-corrected *p* value
The left mOFC–the right mOFC	4.511	0.012
The left lOFC–the left pars orbitalis cortex	4.050	0.032

*OFC* orbitofrontal cortex, *mOFC* medial orbitofrontal cortex, *lOFC* lateral orbitofrontal cortex.

### Structural covariance related to anhedonia in schizophrenia

The stepwise regression analysis showed that structural covariance between the left and right mOFC was positively related to anticipatory pleasure (*B’* = 0.420, *p* = 0.012) and the sum of anticipatory and consummatory pleasure (*B’* = 0.429, *p* = 0.010) (see more details in Table 3).

**Table 3:** Results of stepwise logistic regression analysis.

Pleasure experience	*partial_p*	*B*	SE	*B’*	*t*	*p*	95% CI for *B*	
							Lower	Upper
TEPS-ANT	The left mOFC thickness–right mOFC thickness	2.611	0.982	0.420	2.660	0.012	0.614	4.608
TEPS total score	The left mOFC thickness–right mOFC thickness	4.503	1.652	0.429	2.726	0.010	1.142	7.864

*TEPS-ANT* the anticipatory pleasure subscale of temporal experience of pleasure scale, *TEPS* Temporal Experience of Pleasure Scale, *mOFC* medial orbitofrontal cortex.

## Discussion

It has been proposed that cortical regions, especially the frontal cortex, control higher-order brain functions and exert control over subcortical regions^20,21^. In this study, we investigated the structural covariance abnormalities underlying anhedonia, and there were several major findings. First, compared to healthy controls, patients with schizophrenia manifested both consummatory and anticipatory anhedonia. Second, patients with schizophrenia showed increased structural covariance in cortical thickness. Third, the increased structural covariance between the left and right medial orbitofrontal thickness was negatively correlated with anhedonia in schizophrenia.

### Anhedonia in patients with schizophrenia compared to healthy controls

We found that patients with schizophrenia showed reduced anticipatory and consummatory pleasure, which was congruent with a recent meta-analysis indicating that patients with first-episode or chronic schizophrenia experienced less consummatory and anticipatory pleasure on average than healthy controls^22^. It is assumed that anticipatory pleasure requires cognitive functions that entail predicting future feelings of pleasure. Therefore, anticipatory anhedonia might be associated with cognitive impairments that commonly occur in patients with schizophrenia. In addition, demotivating beliefs in schizophrenia patients with negative symptoms may also impede anticipatory pleasure^23^. Consummatory pleasure is more related to in-the-moment pleasure and is thought to have a strong association with the reward system^24^. However, consummatory pleasure in schizophrenia has long been a debated topic. Many studies have found that consummatory pleasure is intact in schizophrenia patients, even in those with pronounced negative symptoms^25,26^. The inconsistent results from other studies may result from differences in patient characteristics. For example, most of the studies included patients who were administered with antipsychotics. Antipsychotics mostly exert treatment effects by regulating dopaminergic systems, which are closely related to feelings of pleasure and thereby influence the study results.

### Increased structural covariance in cortical thickness in schizophrenia

The regions exhibiting increased structural covariance in the present study included the left and right mOFC, left lateral orbitofrontal cortex and left pars orbitalis cortex.

Schizophrenia typically has an occult onset and exhibits a protracted clinical course. Biological changes, including brain structural and functional alterations, can occur during disease progression or even before illness onset^27–29^. These alterations may relate to the causes, features, or compensatory processes of the disease. Network analysis has shown reorganization patterns of brain function and structure^30–32^ and disturbances in regional correlations such as the frontal regions in schizophrenia^33,34^. Structural changes in brain regions usually appear in a pattern of concomitant loss and thereby exhibit increased structural covariance in schizophrenia^35^. Therefore, we expected a highly organized change in key brain regions. Our observation supports this conjecture.

The specific brain regions involved in the altered structural covariance are promising for providing clues regarding the mechanism of the disease. The OFC is a subdivision of the prefrontal cortex that has been proved to be vital in schizophrenia. It has been reported that the activation of apoptosis in the OFC might contribute to the development of schizophrenia^36^. The OFC volume has also been found to be correlated with negative symptom severity in schizophrenia^37^. In addition, the OFC is responsible for emotional and executive functioning, decision-making, and reward-related behavior, and is associated with emotional disturbances such as social withdrawal, apathy, and depressed mood, which are commonly seen in schizophrenia^38,39^. Regional specificity within the OFC further suggests that the mOFC is activated by emotional stimuli^40^. The lOFC is involved in the process of evaluation, learning of risky bias^41,42^ and emotions such as regret^43^. Although studies on the pars orbitalis are scarce, the role of pars orbitalis in semantic deficits has been demonstrated^44^. The present study suggests that the left and right mOFC, the left lOFC, and the left pars orbitalis were essential regions involved in schizophrenia. Although the literature indicates that these regions might be related to cognitive and emotional disorganization in schizophrenia, longitudinal studies are necessary to fully interpret how these alterations correlate with the characterization, staging, and development of the disease.

### Increased structural covariance in cortical thickness correlated with anhedonia in schizophrenia

As discussed above, we found that schizophrenia patients had significantly lower TEPS score (more severe anhedonia) and increased structural covariance compared to normal controls. What this may indicate is that increased structural covariance may associate with lower TEPS score in schizophrenia patients. However, our further analysis reached the opposite conclusion that the increased structural covariance was positively correlated with higher TEPS score (more mild anhedonia) in these patients. In view of this opposite finding, we reasoned that the phenomenon of increased structural covariance in patients might be a protective effect in patients with schizophrenia against anhedonia. Anhedonia is one of the most important symptoms of schizophrenia and has a high prevalence rate. In biochemical terms, ample evidence supports that dopamine dysregulation is involved in the pathological mechanism of anhedonia^45^. In addition, the association between anhedonia and dopamine dysregulation in the OFC has been reported. Previous positron emission tomography studies recruited healthy volunteers and showed that reward learning processes were correlated with dopamine release in the mOFC and dorsal anterior cingulate cortex (ACC)^46^. Studies including attention deficit hyperactivity disorder or cocaine-dependent subjects also indicated that OFC dysfunction was related to altered dopamine function^47,48^.

On the brain functional and structural level, the OFC is considered to play a crucial role in anhedonia. In major depressive disorder, lower OFC gamma activity was correlated with blunted reward learning^49^, and neural reward prediction error signals in the mOFC were negatively correlated with anhedonia using computational modeling^50^. In schizophrenia, diffusion tensor imaging studies showed that reduced fractional anisotropy in the left posterior mOFC-ACC was associated with anhedonia^39,51^.

In summary, the association between the OFC and anhedonia is multifaceted and interconnected (dopamine function, regional structure and function), although further studies are needed to measure dopamine activity in the OFC. What we found in the structural covariance category has added new evidence for the role of the mOFC in anhedonia in schizophrenia patients. The increased structural covariance between the left mOFC and right mOFC may be a compensatory process for anhedonia in schizophrenia. However, in order to obtain stronger evidence, a longitudinal study design to identify a clear relationship between structural covariance and anhedonia is required.

## Limitations

Several limitations should be addressed here. First, this is a cross-sectional study. Hence, the change in anhedonia, as well as the structural covariance over the stages of schizophrenia, is unknown. Second, we must admit that the evidence of the protective function of increased structural covariance between the left and right mOFC is relatively weak. As such, prospective studies are needed to provide a more convincing result. Third, the present study aimed to reveal the structural covariance between cortical thickness; therefore, the role of cortical-subcortical interaction cannot be determined. Forth, due to the relatively small sample size, we were unable to perform stratified analysis to exclude the influence of the duration of illness and the medication history.

## Conclusion

In the present study, we identified an increased structural covariance mainly in frontal regions, and the increased structural covariance between left and right mOFC might exert a protective effect on anhedonia in schizophrenia.

## Methods

### Subjects

We recruited 80 subjects altogether (35 patients with schizophrenia and 45 healthy volunteers). The inclusion criteria of the schizophrenia group were as follows: (1) met the diagnostic criteria of schizophrenia in the “Diagnostic and Statistical Manual of Mental Disorders, Fourth Edition” (DSM-IV); (2) a minimum education year of 9; (3) was aged from 18 to 50; (3) was Han Chinese and right-handed; (4) did not take antipsychotic medicine within the last 2 weeks; (5) did not have severe somatic diseases such as brain disease; (6) did not have contraindications for MRI; (7) did not receive physical therapy such as transcranial magnetic stimulation or transcranial direct current stimulation within the last 6 months; and (8) did not have any history of psychiatric comorbidity. Schizophrenia patients were recruited from the Shanghai Mental Health Center as outpatients. Every individual was interviewed by two independent and experienced psychiatrists using the “Mini-International Neuropsychiatric Interview” (MINI). We recruited healthy volunteers through advertisement, and each of them was interviewed by an experienced psychiatrist using the MINI. The inclusion criteria for healthy controls were as follows: (1) a minimum education year of 9; (2) aged from 18 to 50; (3) Han Chinese and right-handed; (4) no history or family history of mental illness; (5) no contraindications for MRI; and (6) no severe somatic diseases. All participants were asked to provide written informed consent. The present study was reviewed and approved by the Review Board of the Shanghai Mental Health Center.

### Demographic, clinical, and anhedonic assessments

Demographic information was collected from all participants, including age, sex, education level, duration of illness, marital status, duration of antipsychotic medication, and total antipsychotic dose (olanzapine equivalent) calculated based on defined daily doses. Assessment of clinical symptoms of schizophrenia was performed by trained researchers using the Chinese version of the Positive and Negative Syndrome Scale (PANSS)^52^. The Temporal Experience of Pleasure Scale (Chinese version)^53^ was used to assess anhedonia. The TEPS consists of the anticipatory pleasure subscale (TEPS-ANT) and the consummatory pleasure subscale (TEPS-CON), which reflect anticipatory pleasure and consummatory pleasure, respectively.

### Imaging data acquisition

Brain image data were collected on a 3 Tesla Siemens Prisma magnetic resonance image (MRI) system equipped with a 64-channel radiofrequency coil. All participants underwent high-resolution T1-weighted anatomical imaging. The parameters were: TR = 2000 ms, TE = 2.32 ms, flip = 8°, 208 slices with thickness = 0.9 mm, FOV = 230 mm * 230 mm; and matrix = 256 * 256. Each subject was instructed to lie still in a supine position during scanning.

### Data preprocessing

FreeSurfer v6.0 (https://surfer.nmr.mgh.harvard.edu) was used for imaging preprocessing. The Recon-all command in FreeSurfer was chosen to process whole-brain segmentation automatically. Then each individual’s cortical thickness data were mapped to FreeSurfer’s *fsaverage*. Next, each hemisphere was parcellated into 34 cortical regions using the Desikan-Killiany (DK) atlas^54^, and cortical thickness was measured for 3 subregions (the lateral orbitofrontal, medial orbitofrontal and pars orbitalis) within the OFC.

### Statistical analysis

All statistical analyses were performed in R3.6.3. All tests were two-sided, and the significance level was set as *p* < 0.05. The differences in demographic and clinical information between the schizophrenia group and healthy control group were analyzed using either the independent Student’s *t* test or Pearson’s chi-square test as appropriate. Then, we compared the differences in structural covariance between the two groups. First, we calculated the correlation coefficient between each subregion of the OFC with sex, age, and education level as covariates in the schizophrenia and healthy control groups, respectively. Significant structural covariance in either the schizophrenia or healthy control group was selected for further analysis. Secondly, between-group differences in structural covariance networks within the OFC were assessed using the *z*-test^55^ (see more details in Supplementary Material A). We performed a false discovery rate (FDR) correction for multiple comparisons (FDR < 0.05). The Pearson correlation coefficient (*r*) can be considered the normalized inner product of standard scores (*z* score) as follows:

$${{{\boldsymbol{r}}}} = \frac{1}{{{{{\boldsymbol{N}}}} - 1}}\mathop {\sum}\limits_{{{{\boldsymbol{i}}}} = 1}^{{{\boldsymbol{N}}}} {\left( {\frac{{{{{\boldsymbol{X}}}}_{{{\boldsymbol{i}}}} - {{{\bar{\boldsymbol X}}}}}}{{{{{\boldsymbol{s}}}}_{{{\boldsymbol{X}}}}}}} \right)\left( {\frac{{{{{\boldsymbol{Y}}}}_{{{\boldsymbol{i}}}} - {{{\bar{\boldsymbol Y}}}}}}{{{{{\boldsymbol{s}}}}_{{{\boldsymbol{Y}}}}}}} \right) = \frac{1}{{{{{\boldsymbol{N}}}} - 1}}} \mathop {\sum}\limits_{{{{\boldsymbol{i}}}} = 1}^{{{\boldsymbol{N}}}} {{{{\boldsymbol{z}}}}_{{{{\boldsymbol{X}}}}_{{{\boldsymbol{i}}}}}{{{\boldsymbol{z}}}}_{{{{\boldsymbol{Y}}}}_{{{\boldsymbol{i}}}}}}$$

*X* and *Y* correspond to the thickness of two cortical brain regions across subjects. *N* represents the sample size of each group (the schizophrenia group and healthy control group). $\bar X$ and $\bar Y$ denote the means of *X* and *Y*, respectively. *s* stands for the sample standard deviation. *z*_*Yi*_ and *z*_*Yi*_ equal to $\frac{{{{{\boldsymbol{X}}}}_{{{\boldsymbol{i}}}} - {{{\bar{\boldsymbol X}}}}}}{{{{{\boldsymbol{s}}}}_{{{\boldsymbol{X}}}}}}$ and $\frac{{{{{\boldsymbol{Y}}}}_{{{\boldsymbol{i}}}} - {{{\bar{\boldsymbol Y}}}}}}{{{{{\boldsymbol{s}}}}_{{{\boldsymbol{Y}}}}}}$, respectively. *r* can be regarded as the sum of *partial_p*^56^. *partial_p* can be written as follows:

$${partial\_p} = {{{\boldsymbol{z}}}}_{{{{\boldsymbol{X}}}}_{{{\boldsymbol{i}}}}}{{{\boldsymbol{z}}}}_{{{{\boldsymbol{Y}}}}_{{{\boldsymbol{i}}}}} = \left( {\frac{{{{{\boldsymbol{X}}}}_{{{\boldsymbol{i}}}} - {{{\bar{\boldsymbol X}}}}}}{{{{{\boldsymbol{s}}}}_{{{\boldsymbol{X}}}}}}} \right)\left( {\frac{{{{{\boldsymbol{Y}}}}_{{{\boldsymbol{i}}}} - {{{\bar{\boldsymbol Y}}}}}}{{{{{\boldsymbol{s}}}}_{{{\boldsymbol{Y}}}}}}} \right)$$

Finally, stepwise linear regression analysis was used to explore the relationship between structural covariance and anhedonia in schizophrenia. The structural covariance of any two brain regions that differed significantly was included in the regression analysis. Moreover, illness duration, sex, age, education level, the score of the positive/negative/general psychopathology subscale of the PANSS, duration of antipsychotic medication, and total antipsychotic dose were also included in the regression analysis to exclude their influence as confounding factors.

## Supplementary information

supplemental material
