# Supplementary material for: Increased cortical structural covariance correlates with anhedonia in schizophrenia
Source: Schizophrenia (Heidelb). 2023 Apr 4;9(1):19. doi: 10.1038/s41537-023-00350-3 (PMC10073085; doi:10.1038/s41537-023-00350-3)
Supplement: Supplementary file 1 — supplemental material [file 41537_2023_350_MOESM1_ESM.docx]

A.

Here below is the significant structural covariance in each group:

The schizophrenia group:

The left lOFC-the left mOFC;

The left lOFC-the left pars orbitalis cortex;

The left lOFC-the right lOFC;

The left lOFC-the right mOFC;

the left mOFC-the right lOFC;

the left mOFC-the right pars orbitalis cortex;

the left mOFC- the right mOFC;

the left mOFC-the right lOFC;

the left mOFC-the right pars orbitalis cortex;

the left pars orbitalis cortex - the right pars orbitalis cortex;

the left pars orbitalis cortex- the right lOFC;

the left pars orbitalis cortex- the right mOFC; the right lOFC- the right mOFC

the right lOFC- the right pars orbitalis cortex

The healthy control group:

The left lOFC-the left mOFC;

The left lOFC-the right lOFC;

the right lOFC- the right pars orbitalis cortex;

B.

Here below is R code for between group difference on structural covariance.

library(tidyverse)

library(cocor)

library(psych)

data1 <- data.frame(

v1 = rnorm(50,3,1),

v2 = rnorm(50,7,2),

v3 = rnorm(50,10,3)

)

data2 <- data.frame(

v1 = rnorm(50,6,1),

v2 = rnorm(50,0,2),

v3 = rnorm(50,3,3)

)

cor1 <- corr.test(data1)$r

cor2 <- corr.test(data2)$r

results_z <- data.frame(row.names = colnames(data1))

results_p <- data.frame(row.names = colnames(data1))

for(i in seq_len(nrow(cor1))){

for(j in seq_len(ncol(cor1))){

test <- cocor.indep.groups(cor1[i, j], cor2[i, j], 50, 50) %>% get.cocor.results()

results_z[i, colnames(cor1)[j]] <- test$fisher1925$statistic

results_p[i, colnames(cor1)[j]] <- test$fisher1925$p.value

}

}
